# 355. A Novel Likelihood-Based Model to Estimate SARS-CoV-2 Viral Titer from Next-Generation Sequencing Data

**DOI:** 10.1093/ofid/ofab466.556

**Published:** 2021-12-04

**Authors:** Heather L Wells, Joseph Barrows, Mara Couto-Rodriguez, Xavier O Jirau Serrano, Marilyne Debieu, Karen Wessel, Colleen B Jonsson, Bradley A Connor, Christopher Mason, Dorottya Nagy-Szakal, Niamh B O’Hara

**Affiliations:** 1 Biotia, New York, New York; 2 Labor Zotz/Klimas, Duesseldorf, Nordrhein-Westfalen, Germany; 3 UTHSC, Memphis, Tennessee; 4 Weill Cornell Medicine, New York, New York

## Abstract

**Background:**

The quantitative level of pathogens present in a host is a major driver of infectious disease (ID) state and outcome. However, the majority of ID diagnostics are qualitative. Next-generation sequencing (NGS) is an emerging ID diagnostics and research tool to provide insights, including tracking transmission, evolution, and identifying novel strains.

**Methods:**

We built a novel likelihood-based computational method to leverage pathogen-specific genome-wide NGS data to detect SARS-CoV-2, profile genetic variants, and furthermore quantify levels of these pathogens. We used de-identified clinical specimens tested for SARS-CoV-2 using RT-PCR, SARS-CoV-2 NGS Assay (hybrid capture, Twist Bioscience), or ARTIC (amplicon-based) platform, and COVID-DX software. A training (n=87) and validation (n=22) set was selected to establish the strength of our quantification model. We fit non-uniform probabilistic error profiles to a deterministic sigmoidal equation that more realistically represents observed data and used likelihood maximized over several different read depths to improve accuracy over a wide range of values of viral load. Given the proportion of the genome covered at varying depths for a single sample as input data, our model estimated the Ct of that sample as the value that produces the maximum likelihood of generating the observed genome coverage data.

**Results:**

The model fit on 87 SARS-CoV-2 NGS Assay training samples produced a good fit to the 22 validation samples, with a coefficient of correlation (r2) of ~0.8. The accuracy of the model was high (mean absolute % error of ~10%, meaning our model is able to predict the Ct value of each sample within a margin of ±10% on average). Because of the nature of the commonly used ARTIC protocol, we found that all quantitative signals in this data were lost during PCR amplification and the model is not applicable for quantification of samples captured this way. The ability to model quantification is a major advantage of the SARS-CoV-2 NGS assay protocol.

The likelihood-based model to estimate SARS-CoV-2 viral titer

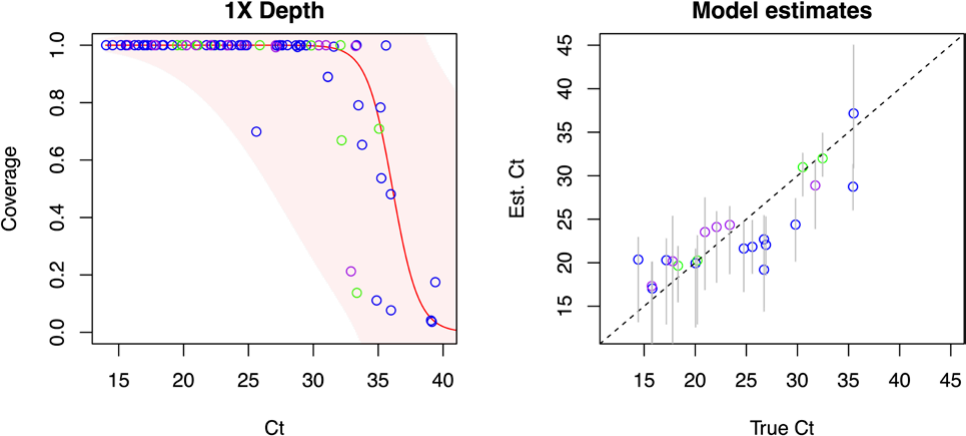

**Left:**

Observed genome coverage (y-axis) plotted against Ct value (x-axis). The best-fitting logistic curve is demonstrated with a red line with shaded areas above and below representing the fitted error profile. RIGHT: Model-estimated Ct values (y-axis) compared to laboratory Ct values (x-axis) with grey bars representing estimated confidence intervals. The 1:1 diagonal is shown as a dotted line.

**Conclusion:**

To our knowledge, this is the first model to incorporate sequence data mapped across the genome of a pathogen to quantify the level of that pathogen in a clinical specimen. This has implications in ID diagnostics, research, and metagenomics.

**Disclosures:**

**Heather L. Wells, MPH**, **Biotia, Inc.** (Consultant) **Joseph Barrows, MS**, **Biotia** (Employee) **Mara Couto-Rodriguez, MS**, **Biotia** (Employee) **Xavier O. Jirau Serrano, B.S.**, **Biotia** (Employee) **Marilyne Debieu, PhD**, **Biotia** (Employee) **Karen Wessel, PhD**, **Labor Zotz/Klimas** (Employee) **Christopher Mason, PhD**, **Biotia** (Board Member, Advisor or Review Panel member, Shareholder) **Dorottya Nagy-Szakal, MD PhD**, **Biotia Inc** (Employee, Shareholder) **Niamh B. O’Hara, PhD**, **Biotia** (Board Member, Employee, Shareholder)

